# Digital Manufacturing and Periodontal Performance of CAD/CAM-Customized Orthodontic Molar Bands Compared with Standard Stainless-Steel Bands

**DOI:** 10.3390/medicina62050967

**Published:** 2026-05-15

**Authors:** Sorana Maria Bucur, Clara Diana Haddad, Loredana Mițariu, Mihai Mițariu, Mariana Păcurar

**Affiliations:** 1 Department of Dentistry, Faculty of Medicine, “Dimitrie Cantemir” University of Târgu Mureș, 3–5 Bodoni Sandor Str., 540545 Târgu-Mureș, Romania; bucursoranamaria@gmail.com; 2 Department of Orthodontics, Faculty of Dentistry, “Grigore T. Popa” University of Medicine and Pharmacy Iași, 700115 Iași, Romania; 3 Department IV of Dental Medicine and Nursing, Faculty of Dentistry, University of Sibiu “Lucian Blaga” (ULBS), Lucian Blaga 2A, 550169 Sibiu, Romania; loredana.mitariu@ulbsibiu.ro; 4 Department of Orthodontics, Faculty of Dental Medicine, “George Emil Palade” University of Medicine and Pharmacy, Sciences and Technology, 540139 Târgu-Mureș, Romania; mariana.pacurar@umfst.ro

**Keywords:** CAD/CAM manufacturing, orthodontic bands, periodontal health, digital dentistry, stainless-steel biomaterials, orthodontic appliances

## Abstract

*Background and Objectives*: Conventional stainless-steel orthodontic molar bands may exhibit limited anatomical adaptation, favoring plaque retention and periodontal inflammation. This study aimed to compare the periodontal outcomes of standard bands and CAD/CAM-customized molar bands in adolescents. *Materials and Methods*: A prospective randomized controlled clinical study was conducted in 180 adolescents (mean age: 11.9 years) undergoing fixed orthodontic therapy. Participants were allocated to CAD/CAM-customized bands (*n* = 90) or standard stainless-steel bands (n = 90). Periodontal parameters—Plaque Control Record (PCR), Bleeding on Probing (BOP), and Periodontal Probing Depth (PPD)—were assessed at baseline, 1, 3, and 6 months. Data were analyzed using the Mann–Whitney U test (*p* < 0.05). *Results*: Baseline values were comparable between groups (*p* > 0.05). During follow-up, the CAD/CAM group showed significantly lower PCR, BOP, and PPD values. At 6 months, PCR was 21 ± 8% vs. 42 ± 12%, BOP was 17 ± 6% vs. 40 ± 10%, and PPD was 2.5 ± 0.5 mm vs. 3.1 ± 0.6 mm (all *p* < 0.001). *Conclusions*: CAD/CAM-customized molar bands demonstrated superior periodontal performance compared with conventional bands. Improved anatomical adaptation may reduce plaque accumulation and gingival inflammation during orthodontic treatment.

## 1. Introduction

Orthodontic molar bands are traditionally manufactured from stainless-steel alloys due to their high mechanical strength, corrosion resistance, and established biocompatibility in the oral environment [1,2]. These bands remain an essential component of fixed orthodontic appliances, providing anchorage for auxiliary devices such as palatal expanders, headgear, and various traction systems [1]. Despite their widespread use and favorable mechanical properties, conventional prefabricated molar bands often present limitations related to their standardized geometry. Because these bands are produced in predefined sizes, they may not precisely conform to the anatomical morphology of individual molars.

Inadequate adaptation between the band and the tooth surface may create marginal discrepancies and microgaps that favor bacterial biofilm retention. These areas can act as plaque-retentive niches, increasing the risk of gingival inflammation, bleeding on probing, and localized periodontal changes during orthodontic treatment [2,3]. Such periodontal responses are particularly relevant in adolescent patients undergoing fixed orthodontic therapy, as orthodontic appliances can complicate routine oral hygiene procedures and facilitate plaque accumulation around bands and brackets.

**Figure 1:**
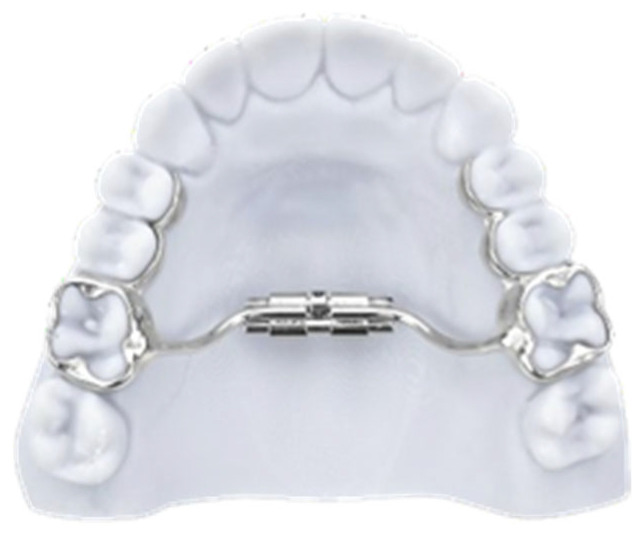
Example of molar band placement in a fixed orthodontic appliance.

Recent advances in digital dentistry have introduced new possibilities for the design and fabrication of orthodontic appliances. Computer-aided design and computer-aided manufacturing (CAD/CAM) technologies integrate intraoral scanning, digital modeling, and high-precision manufacturing processes to produce patient-specific dental components (Figure 1).

Digital workflows are widely adopted in prosthodontics and implant dentistry, where they have demonstrated improved marginal accuracy and reproducibility compared with conventional fabrication techniques. In orthodontics, however, the application of CAD/CAM technologies to molar band design remains relatively limited [4,5,6].

Customized CAD/CAM-manufactured molar bands offer the potential advantage of precise anatomical adaptation to the tooth surface. By reproducing the exact morphology of the molar crown, digitally designed bands may reduce marginal discrepancies at the band–tooth interface, thereby minimizing plaque-retentive areas and improving periodontal compatibility during orthodontic treatment [5,7]. Preliminary investigations have suggested that such customization may contribute to reduced plaque accumulation and improved gingival health. Nevertheless, most available studies evaluating digitally manufactured orthodontic appliances have involved relatively small patient cohorts, often including fewer than 30–40 participants, which limits the statistical power and generalizability of the findings [5,7].

Therefore, a larger-scale clinical evaluation is necessary to better understand the periodontal implications of customized orthodontic band design. A more robust sample size allows for more reliable statistical analysis and provides stronger evidence for clinical decision-making regarding appliance selection.

The present prospective clinical study was designed to compare periodontal outcomes associated with conventional preformed stainless-steel molar bands and CAD/CAM-customized bands in a cohort of 180 adolescents undergoing fixed orthodontic therapy. Periodontal parameters, including Plaque Control Record (PCR), Bleeding on Probing (BOP), and Periodontal Probing Depth (PPD), were evaluated over a six-month observation period to assess plaque accumulation, gingival inflammation, and early periodontal changes associated with each band design.

The null hypothesis of this study was that no significant differences would exist between conventional preformed molar bands and CAD/CAM-customized bands regarding plaque accumulation, gingival bleeding, and probing depth progression during orthodontic treatment.

## 2. Materials and Methods

### 2.1. Study Design and Participants

This study was designed and reported in accordance with the CONSORT guidelines for randomized controlled trials. A total of 180 patients in the mixed and permanent dentition stages (mean age: 11.9 ± 2.6 years; 108 boys and 72 girls) undergoing fixed orthodontic therapy at the Dental Clinic of the “Dimitrie Cantemir” University of Târgu Mureș between 2024 and 2025 were enrolled.

A priori power analysis indicated that at least 80 patients per group were required to detect a minimum difference of 10% in Plaque Control Record (PCR) values between groups with a statistical power of 0.80 and a significance level of 0.05.

The study protocol was approved by the “Dimitrie Cantemir” University Ethics Committee (Decision No. 18/18 March 2024). Written informed consent was obtained from all participants and their legal guardians before inclusion in the study.

The study was completed as planned after the six-month follow-up period for all participants. No interim analyses, stopping rules, or early termination criteria were applied.

This clinical trial was registered at ClinicalTrials.gov (Identifier: NCT07526805) on 7 April 2026. The study completion date was 26 February 2026. The registration was performed retrospectively after participant enrollment had begun. The trial was not prospectively registered because the study was initially designed as an institutional clinical investigation with no intention of submission to an international trial registry. The authors subsequently registered the study to ensure transparency and compliance with current reporting standards. The study protocol, methodology, and outcome measures were defined before participant enrollment and were not modified after study initiation.

### 2.2. Randomization Procedure

Participants were randomly allocated to one of two treatment groups using computer-generated random numbers produced with a block randomization algorithm (block size = 10) to ensure balanced group distribution throughout the recruitment period. Allocation sequences were generated by an independent researcher who was not involved in clinical procedures or outcome assessment.

Assignments were placed in sealed opaque envelopes, which were opened only at the time of appliance placement, ensuring allocation concealment.

Participants were assigned to:

Group A—CAD/CAM-customized bands (*n* = 90). Bands were fabricated using digital intraoral scans, computer-aided design software, and high-precision milling, followed by thermal sintering to achieve individualized anatomical adaptation.

Group B—Standard preformed bands (*n* = 90).

Conventional stainless-steel orthodontic bands were selected from commercially available size kits and manually adapted to the molars.

Inclusion and Exclusion Criteria

Inclusion criteria:

- Adolescents requiring fixed orthodontic therapy involving molar bands;
- Good general health with no systemic diseases affecting periodontal tissues (e.g., diabetes mellitus, chronic kidney disease);
- Acceptable baseline oral hygiene;
- Patient compliance with follow-up visits.

Exclusion criteria:

- History of periodontal disease;
- Current antibiotic or anti-inflammatory therapy;
- Dental trauma affecting first molars;
- Poor oral hygiene compliance during screening.

All randomized participants (*n* = 180) completed the study and were included in the final analysis. No losses to follow-up, exclusions, or withdrawals occurred during the study period. Therefore, a complete-case analysis was performed, and no imputation methods were required.

### 2.3. Digital Design and Manufacturing

Digital impressions were obtained using an intraoral scanner (Trios 3, 3Shape, Copenhagen, Denmark). Band geometry was designed using specialized CAD software (3Shape Dental System 2023.1, 3Shape, Copenhagen, Denmark) to reproduce the exact anatomical morphology of the molar crown. CAD/CAM-customized bands were manufactured from medical-grade cobalt–chromium (Co–Cr) alloy using high-precision milling followed by thermal sintering.

#### Cementation Protocol and Blinding

A single experienced orthodontist cemented all orthodontic bands to ensure consistency of clinical procedures. Dual-cure Unitek™ (3M Unitek, Monrovia, CA, USA) Multi-Cure Glass Ionomer cement was used according to the manufacturer’s instructions. Apart from the manufacturing method and geometric customization of the bands, all other clinical procedures were identical between the two study groups. Both CAD/CAM-customized and standard stainless-steel bands were cemented using the same glass ionomer cement, applied by the same operator, and incorporated into identical fixed orthodontic appliance systems. The follow-up schedule, oral hygiene instructions, and periodontal assessment protocol were standardized for all participants. This ensured that the type of molar band represented the only experimental variable, thereby minimizing potential performance and detection bias between interventions.

Excess cement was carefully removed to minimize subgingival remnants.

Because the operator placing the bands necessarily knew the appliance type, operator blinding was not feasible during cementation. However, the examiner responsible for periodontal measurements was blinded to group allocation, reducing measurement bias.

### 2.4. Clinical Periodontal Measurements

Periodontal parameters were assessed using standardized clinical indices:

Plaque Control Record (PCR):

Plaque presence was recorded on four molar surfaces (mesial, distal, vestibular, and palatal).

Bleeding on Probing (BOP):

Recorded at six sites per molar (three vestibular and three palatal) following gentle periodontal probing.

Periodontal Probing Depth (PPD):

Measured using a calibrated Williams periodontal probe. Values greater than 3 mm were considered indicative of early periodontal inflammation.

Measurements were recorded at:

- Baseline (T0);
- One month (T1);
- Three months (T2);
- Six months (T3),

No adverse events or treatment-related harms were observed or systematically recorded during the study period.

### 2.5. Examiner Calibration and Reproducibility

All periodontal measurements were performed by a single calibrated examiner who was blinded to treatment allocation.

To evaluate measurement reproducibility, repeated periodontal measurements were performed in 20 randomly selected patients within a two-week interval. The intraclass correlation coefficient (ICC) was 0.91, indicating excellent intra-examiner reliability.

### 2.6. Statistical Analysis

Statistical analyses were performed using Python version 3.11 (Python Software Foundation, Wilmington, DE, USA) with the Pandas 2.2.2, SciPy 1.13.1, and Seaborn 0.13.2 libraries.

Normality was assessed using the Shapiro–Wilk test, which indicated a non-normal distribution for PCR, BOP, and PPD values. Because the study aimed to compare intergroup differences at each time point, Mann–Whitney U tests were applied independently for T1, T2, and T3 comparisons.

Effect size was calculated using Cohen’s d to estimate the magnitude of differences between groups.

Statistical significance was set at *p* < 0.05.

## 3. Results

This study evaluated 180 adolescents undergoing fixed orthodontic therapy with either CAD/CAM-customized or standard preformed molar bands. Periodontal outcomes were assessed at baseline (T0), 1 month (T1), 3 months (T2), and 6 months (T3) using PCR, BOP, and PPD (Table 1, Table 2 and Table 3) between 2024 and 2025. The data showed consistently superior periodontal outcomes in the CAD/CAM group, with lower plaque accumulation, reduced gingival bleeding, and shallower probing depths at all follow-up points.

**Figure 2:**
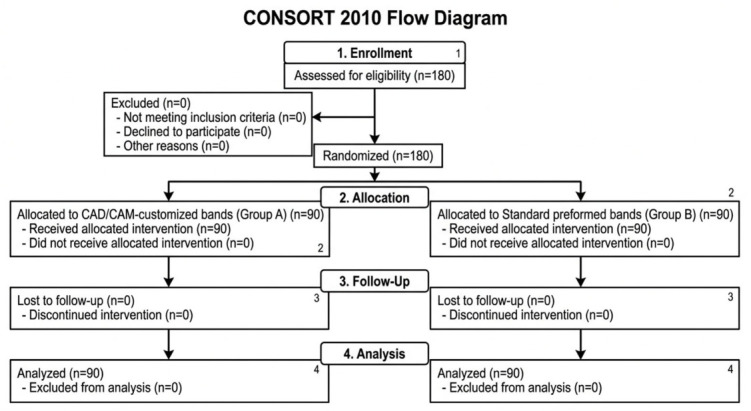
CONSORT flow diagram of participant recruitment, allocation, follow-up, and analysis.

The flow of participants through each stage of the study is presented in Figure 2. All randomized participants completed the study, and no losses to follow-up or exclusions occurred.

### 3.1. Plaque Control Record (PCR)

At baseline, PCR values were comparable between groups. By 1 month, the standard band group showed a significant increase in plaque accumulation, which persisted at 3 and 6 months. In contrast, the CAD/CAM group maintained low PCR values throughout the observation period, indicating superior plaque control.

### 3.2. Bleeding on Probing (BOP)

CAD/CAM-customized bands were associated with significantly lower gingival bleeding at all time points. The standard band group exhibited progressive increases in BOP, peaking at 40% at 6 months, reflecting a higher inflammatory response.

### 3.3. Periodontal Probing Depth (PPD)

Interpretation:

The CAD/CAM group maintained shallower periodontal pockets, suggesting reduced inflammatory progression and better preservation of periodontal support. Standard bands were associated with increased PPD, reflecting early tissue compromise. Shapiro–Wilk tests confirmed a non-normal distribution for PCR, BOP, and PPD, justifying the use of Mann–Whitney U tests for between-group comparisons. Mann–Whitney U testing confirmed significant differences between groups at T2 and T3 (*p* < 0.001). All three periodontal indices showed statistically significant differences in favor of CAD/CAM bands at 1, 3, and 6 months (*p* < 0.01 for PCR and BOP at 1 month; *p* < 0.001 at 3 and 6 months).

Trends over time:

1. PCR: The CAD/CAM group remained near baseline (~15–21%), whereas the standard group increased from 16% to 42% by 6 months.
2. BOP: Gingival inflammation increased modestly in the CAD/CAM group (12% → 17%) but doubled in the standard group (13% → 40%).
3. PPD: CAD/CAM bands showed minimal probing depth increases (2.2 → 2.5 mm), while standard bands increased from 2.3 to 3.1 mm, representing early periodontal compromise.

**Figure 3:**
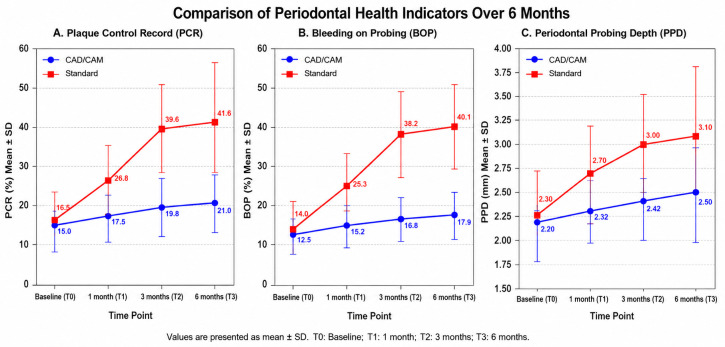
Combined evolution of periodontal parameters (PCR, BOP, and PPD) in patients treated with CAD/CAM-customized and standard orthodontic molar bands during the 6-month observation period.

These trends indicate that customized anatomical adaptation of molar bands reduces plaque accumulation, gingival bleeding, and periodontal pocket formation, supporting the clinical advantage of CAD/CAM bands. The temporal evolution of the principal periodontal parameters recorded during orthodontic treatment is illustrated in Figure 3. The graphical representation highlights the progressive increase observed in Plaque Control Record (PCR), Bleeding on Probing (BOP), and Periodontal Probing Depth (PPD) throughout the monitoring intervals, reflecting the cumulative influence of orthodontic band placement on periodontal status.

## 4. Discussion

The present prospective clinical study compared periodontal outcomes associated with CAD/CAM-customized orthodontic molar bands and conventional preformed stainless-steel bands in a cohort of 180 adolescents undergoing fixed orthodontic therapy. The results demonstrated that digitally manufactured bands were associated with significantly improved periodontal parameters over the six-month observation period, including lower plaque accumulation, reduced gingival bleeding, and smaller increases in periodontal probing depth [5,7]. These findings suggest that the improved anatomical adaptation achieved through digital manufacturing may contribute to better preservation of periodontal health during orthodontic treatment [8].

From a biomaterials perspective, orthodontic molar bands are typically produced from austenitic stainless-steel alloys because of their favorable mechanical strength, corrosion resistance, and long-term biocompatibility in the oral environment [9]. These alloys generally contain chromium and nickel, which promote the formation of a protective chromium oxide layer that limits corrosion and metal ion release in the oral cavity [10,11]. However, the clinical performance of orthodontic appliances depends not only on material composition but also on the accuracy of the interface between the appliance and dental tissues [12]. Even when biocompatible materials are used, inadequate adaptation of orthodontic bands can create marginal irregularities that favor bacterial colonization and biofilm accumulation. No adverse events or treatment-related complications were observed or systematically documented throughout the study period.

The present findings indicate that CAD/CAM-customized bands provide superior anatomical conformity to the tooth surface compared with conventional prefabricated bands. This improved adaptation appears to reduce plaque-retentive microgaps at the band–tooth interface. In the current study, Plaque Control Record values remained relatively stable in the CAD/CAM group, increasing only slightly during follow-up, whereas the standard band group showed a progressive increase in plaque accumulation. These results support the hypothesis that precise digital customization may limit bacterial biofilm retention on orthodontic appliances [2].

A similar pattern was observed for gingival inflammation. Bleeding on probing values increased considerably in the standard band group but remained comparatively low in the CAD/CAM group throughout the observation period. Because bacterial biofilm represents the principal etiological factor for gingival inflammation, the improved plaque control associated with customized bands likely contributed to the lower inflammatory response observed in these patients [13,14].

Periodontal probing depth measurements further support this interpretation. Although slight increases in probing depth were observed in both groups during orthodontic treatment, the increase was significantly smaller in the CAD/CAM group. The probing depths recorded in the present study remained within the range typically associated with reversible gingival inflammation rather than established periodontal disease. Nevertheless, the smaller increase observed in the customized band group suggests that improved marginal adaptation may reduce early periodontal changes during orthodontic therapy [2,15].

These clinical findings are consistent with the manufacturing advantages provided by digital dentistry workflows. CAD/CAM technologies combine intraoral scanning, computer-aided design, and high-precision manufacturing techniques such as milling or sintering to produce patient-specific orthodontic components [5,7]. In contrast to conventional prefabricated bands, which require manual chairside adaptation, digitally designed bands replicate the morphology of the molar crown with greater dimensional accuracy. Improved continuity at the band–tooth interface may therefore reduce surface irregularities that act as microbial retention sites [5,7,16].

Previous research investigating periodontal changes during orthodontic treatment has consistently reported increased plaque accumulation and gingival inflammation associated with fixed appliances, particularly around molar bands and brackets. However, most studies evaluating the periodontal effects of orthodontic appliances have involved relatively small patient populations and rarely focused on digitally manufactured band designs [17,18]. The relatively large cohort included in the present study strengthens the statistical reliability of the findings and provides additional evidence supporting the periodontal advantages of customized orthodontic appliances.

Despite these encouraging results, several limitations should be considered when interpreting the findings. First, the observation period was limited to six months, which allowed evaluation of early periodontal responses but does not fully represent the entire duration of orthodontic therapy, which often extends over multiple years [19]. Longer follow-up studies are therefore necessary to determine whether the periodontal benefits observed with CAD/CAM-customized bands persist throughout prolonged treatment periods.

Second, the study focused primarily on clinical periodontal parameters, including plaque accumulation, bleeding on probing, and probing depth. While these indices are widely accepted indicators of periodontal health, microbiological analysis of the bacterial biofilm associated with orthodontic bands was not performed. Future investigations incorporating microbial profiling could provide deeper insight into how band design influences bacterial colonization and biofilm composition.

Third, patient-reported outcomes such as comfort, ease of oral hygiene maintenance, and perceived appliance stability were not evaluated in the present study. These parameters may influence patient compliance and treatment satisfaction and should therefore be explored in future clinical research.

From a clinical perspective, the results of this study suggest that the integration of digital manufacturing technologies into orthodontic band fabrication may offer practical advantages for both clinicians and patients. CAD/CAM-customized bands may reduce the need for extensive chairside band adaptation and improve the predictability of appliance fit [5,7]. Improved periodontal compatibility may also help reduce gingival inflammation in adolescents undergoing orthodontic treatment, a population in which maintaining optimal oral hygiene can be challenging [18,20,21,22].

Although digital manufacturing may initially involve higher laboratory costs compared with conventional prefabricated bands, these costs may be partially offset by reductions in chairside adjustment time and potential improvements in periodontal health during treatment. As digital workflows become increasingly accessible in orthodontic practice, customized appliance fabrication may represent a clinically viable approach for improving the biological compatibility of orthodontic devices [23,24].

Overall, the findings of the present study highlight the importance of appliance design and manufacturing precision in maintaining periodontal health during orthodontic treatment. By combining well-established stainless-steel biomaterials with advanced digital manufacturing techniques, CAD/CAM-customized molar bands represent a promising development in orthodontic appliance design and personalized dental care.

Additionally, patient-reported outcomes such as comfort, satisfaction, and ease of oral hygiene maintenance were not evaluated in the present study. These parameters are clinically relevant, as they may influence patient compliance and the overall treatment experience. Furthermore, the study population consisted exclusively of adolescent patients undergoing orthodontic treatment. As periodontal response and oral hygiene behavior may differ in adult populations, the generalizability of these findings to other age groups may be limited. Future research should include a broader range of age groups to validate the applicability of these results across different patient populations.

Future studies should incorporate validated patient-reported outcome measures to provide a more comprehensive assessment of the clinical performance of orthodontic bands. They should also focus on long-term clinical outcomes, microbiological analysis of orthodontic biofilms, and the mechanical and corrosion behavior of digitally manufactured orthodontic bands. Multicenter clinical trials involving diverse patient populations would also help further validate the clinical advantages of CAD/CAM-customized orthodontic appliances.

From an economic perspective, CAD/CAM-customized orthodontic bands may involve higher initial laboratory and manufacturing costs compared with conventional prefabricated stainless-steel bands. These additional costs are related to digital scanning, software design, and precision manufacturing processes [25]. However, improved anatomical fit may reduce chairside adjustment time and potentially decrease the risk of periodontal complications, which could translate into long-term clinical and economic benefits. Future cost–effectiveness analyses are needed to better evaluate the balance between the initial investment and the potential clinical advantages.

## 5. Conclusions

The present study demonstrates that CAD/CAM-customized orthodontic molar bands provide significantly better periodontal outcomes than conventional preformed stainless-steel bands in adolescent patients undergoing fixed orthodontic therapy. Improved anatomical adaptation achieved through digital manufacturing appears to reduce plaque accumulation, gingival bleeding, and increases in probing depth during treatment.

These findings suggest that the integration of CAD/CAM technology into orthodontic band fabrication may enhance periodontal compatibility and contribute to improved oral health during orthodontic treatment. Further long-term and multicenter investigations incorporating microbiological analysis and patient-reported outcomes are recommended to fully evaluate the clinical advantages of digitally manufactured orthodontic bands.

## Figures and Tables

**Table 1:** Mean Plaque Control Record (PCR) (%) at baseline and follow-up for CAD/CAM and standard bands.

Time Point	CAD/CAM PCR (%) Mean ± SD	Standard PCR (%) Mean ± SD	*p*-Value
Baseline (T0)	15 ± 5	16 ± 6	0.67
1 month (T1)	18 ± 6	27 ± 8	<0.01
3 months (T2)	20 ± 7	40 ± 10	<0.001
6 months (T3)	21 ± 8	42 ± 12	<0.001

**Table 2:** Mean Bleeding on Probing (BOP) (%) at baseline and follow-up for CAD/CAM and standard bands.

Time Point	CAD/CAM BOP (%) Mean ± SD	Standard BOP (%) Mean ± SD	*p*-Value
Baseline (T0)	12 ± 4	13 ± 5	0.54
1 month (T1)	14 ± 5	25 ± 7	<0.01
3 months (T2)	16 ± 6	38 ± 9	<0.001
6 months (T3)	17 ± 6	40 ± 10	<0.001

**Table 3:** Mean Periodontal Probing Depth (PPD) (mm) at baseline and follow-up for CAD/CAM and standard bands.

Time Point	CAD/CAM PPD (mm) Mean ± SD	Standard PPD (mm) Mean ± SD	*p*-Value
Baseline (T0)	2.2 ± 0.3	2.3 ± 0.4	0.62
1 month (T1)	2.3 ± 0.4	2.7 ± 0.5	<0.01
3 months (T2)	2.4 ± 0.4	3.0 ± 0.5	<0.001
6 months (T3)	2.5 ± 0.5	3.1 ± 0.6	<0.001

## Data Availability

Data supporting the findings of this study are available from the corresponding author upon reasonable request.
